# Molecular Docking and* In Silico* ADMET Study Reveals Acylguanidine 7a as a Potential Inhibitor of *β*-Secretase

**DOI:** 10.1155/2016/9258578

**Published:** 2016-04-10

**Authors:** Chaluveelaveedu Murleedharan Nisha, Ashwini Kumar, Prateek Nair, Nityasha Gupta, Chitrangda Silakari, Timir Tripathi, Awanish Kumar

**Affiliations:** ^1^Department of Biotechnology, National Institute of Technology, Raipur, Chhattisgarh 492010, India; ^2^Molecular and Structural Biophysics Laboratory, Department of Biochemistry, North Eastern Hill University, Shillong 793022, India

## Abstract

Amyloidogenic pathway in Alzheimer's disease (AD) involves breakdown of APP by *β*-secretase followed by *γ*-secretase and results in formation of amyloid beta plaque. *β*-secretase has been a promising target for developing novel anti-Alzheimer drugs. To test different molecules for this purpose, test ligands like acylguanidine 7a, rosiglitazone, pioglitazone, and tartaric acid were docked against our target protein *β*-secretase enzyme retrieved from Protein Data Bank, considering MK-8931 (phase III trial, Merck) as the positive control. Docking revealed that, with respect to their free binding energy, acylguanidine 7a has the lowest binding energy followed by MK-8931 and pioglitazone and binds significantly to *β*-secretase.* In silico* ADMET predictions revealed that except tartaric acid all other compounds had minimal toxic effects and had good absorption as well as solubility characteristics. These compounds may serve as potential lead compound for developing new anti-Alzheimer drug.

## 1. Introduction

Alzheimer's disease (AD), the irreversible and progressive disease of the brain, is one of the most common causes of dementia in our society which gradually destroys our cognitive ability [1]. Alzheimer's Association says it accounts for between 60% and 80% of all cases of dementia, occurring primarily in people above 60 years of age [2]. It is characterized by acceleration of amyloid *β* (A*β*) plaque accumulation around neurons and hyperphosphorylation of tau leading to the accumulation of neurofibrillary tangles (NFTs) within brain cells [3]. Furthermore, degradation of hyperphosphorylated tau by the proteasome is inhibited by the actions of A*β*. Amyloidogenic pathway is the result of a mutation that replaces the normal pathway in which *α*-secretase acts on the APP followed by *γ*-secretase forming harmless p-3 peptide but the amyloidogenic pathway involves breakdown of APP by *β*-secretase followed by *γ*-secretase and results in formation of amyloid beta plaque [4, 5].

**Figure 1:**
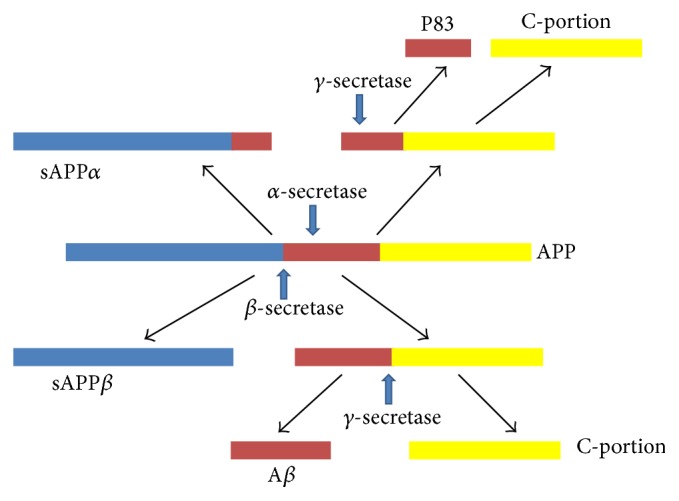
The APP processing (in brief): APP: amyloid precursor protein; sAPP: soluble APP fraction; A*β*: amyloid beta; C-portion: C-terminal portion obtained from final cleavage.

The extracellular domain of *β*-amyloid precursor protein (APP) undergoes proteolytic cleavage by *β*-site APP cleaving enzyme (BACE 1 or *β*-secretase) followed by cleavage of the transmembrane domain of *β*-APP by *γ*-secretase. With all these cleavage sites and several peptides being produced, it is becoming more apparent that other APP-derived peptides beyond A*β* also may play critical roles in AD phenotype [6, 7]. Therefore, targeting BACE 1 enzyme could be useful in controlling the formation and appearance of the pathogenic amyloid *β* peptides. BACE 1 enzyme may hold a surprising central position. This is because cleavage of APP by BACE 1 not only generates the C-terminal fragment of APP that is the direct precursor of A*β* but also releases sAPP*β*, which can interact with DR6 to effect neuronal damage. The *γ*-secretase cleavage of APP produces a fragment known as P83 after *α*-secretase pathway while it produces neurotoxic A*β* after *β*-secretase pathway [4]. Another important mechanism involving A*β* mediated neuronal inflammation and gradual cell death is their interaction with Receptors for Advanced Glycation End Products (RAGE) found on neurons and astrocytes [8], Figure 1.

Our study involves docking of *β*-secretase with four compounds, namely, acylguanidine 7a, tartaric acid, rosiglitazone, and pioglitazone, and comparison of these results with docking results of MK-8931 (as positive control) with *β*-secretase. Acylguanidine 7a, TZDs (thiazolidinediones), and tartaric acid were selected randomly based on a few previous studies [9–13]. MK-8931 is an anti-AD BACE 1 inhibitor candidate of Merck Inc. that has entered phase III clinical trials [14].

## 2. Materials and Method

### 2.1. Materials

Docking is a computational simulation approach of a candidate ligand binding to a receptor and predicts the preferred orientation of binding of one molecule to the second to form a stable complex. Docking is used to predict the affinity and activity of binding of the small molecule to their protein targets by using scoring functions. Hence, docking plays an important role in the rational design of drugs. The sensitivity of docking calculations regarding the geometry of the input ligand shows that even small changes in the ligand conformation can lead to large differences in the geometries and scores of the resulting docked poses.

Here, we worked with web-based online molecular docking program Docking Server. Drug-likeness was calculated with OSIRIS Property Explorer while the ADMET profiling was done with admetSAR program. The program was able to calculate the essential docking parameters with satisfactory results. It provided us with a detailed docking result against which we can determine the effectiveness of the test ligand.

### 2.2. Selection of Ligand

**Figure 2:**
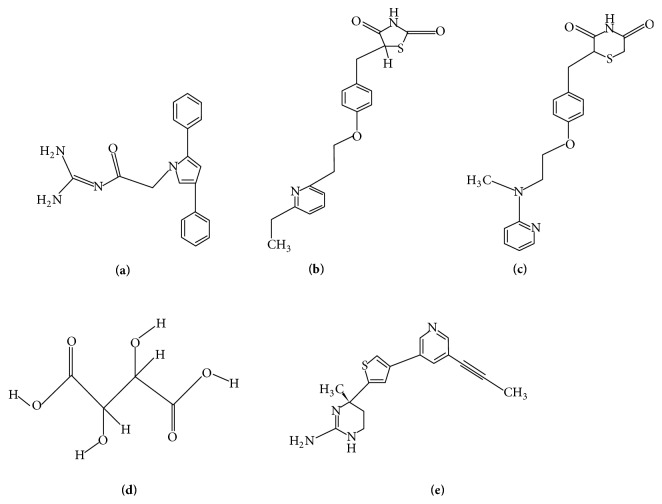
Test ligands (**a**)–(**e**): acylguanidine 7a (**a**), pioglitazone (**b**), rosiglitazone (**c**), tartaric acid (**d**), and MK-8931 (control,** e**).

Analogs of acylguanidine 7a, rosiglitazone, pioglitazone, and tartaric acid were identified as potential *β*-secretase inhibitors from different literature reviews. Their structures were drawn afresh using the software* ChemSketch (ACD/Labs, v12.01)*. Figures 2(**a**)–(**e**) show the structures of different ligands including the control compound MK-8931 from Merck.

### 2.3. Selection of Receptor

The X-ray crystal coordinates of *β*-secretase (BACE 1) (PDB ID: 1SGZ) were retrieved from* Protein Data Bank* (http://www.rcsb.org/pdb/home/home.do). Since *β*-secretase has its crystal structure in a state that represents the pharmacological target for the development of new drugs to cure AD, it is selected for modeling studies. PDBsum (http://www.ebi.ac.uk/thornton-srv/databases/cgi-bin/pdbsum/GetPage.pl?pdbcode=index.html) server was used to determine the active sites of receptor and determine their interactions with compounds. BACE 1 is an aspartyl protease (Figure 2).

### 2.4. Molecular Docking

Docking Server (http://www.dockingserver.com/web) is a web-based, easy-to-use interface that handles all aspects of molecular docking from ligand and protein setup. It also provides full control on the setting of specific parameters of ligand and protein setup and docking calculations for more advanced users. It allows the user to carry out highly efficient and robust docking calculations by integrating a number of popular software programs used in* in silico* chemistry into one comprehensive web service.

### 2.5. Drug-Likeness Prediction

The OSIRIS Property Explorer uses chemical structures and calculates on-the-fly various drug-relevant properties whenever a structure is valid. Prediction results are valued and color coded. Properties analyzed are TPSA, *c*log⁡*P* calculation, log⁡*S* calculation, molecular weight, fragment based drug-likeness, and drug score.

### 2.6. ADMET Prediction

ADMET properties of a compound deal with its absorption, distribution, metabolism, excretion, and toxicityin and through the human body. ADMET, which constitutes the pharmacokinetic profile of a drug molecule, is very essential in evaluating its pharmacodynamic activities. Today a lot of online tools and offline software programs are available which helps us in predicting this behaviour of the drug candidate. In this study, we have used the admetSAR prediction tool (http://lmmd.ecust.edu.cn:8000/).

## 3. Results and Discussion

**Figure 3:**
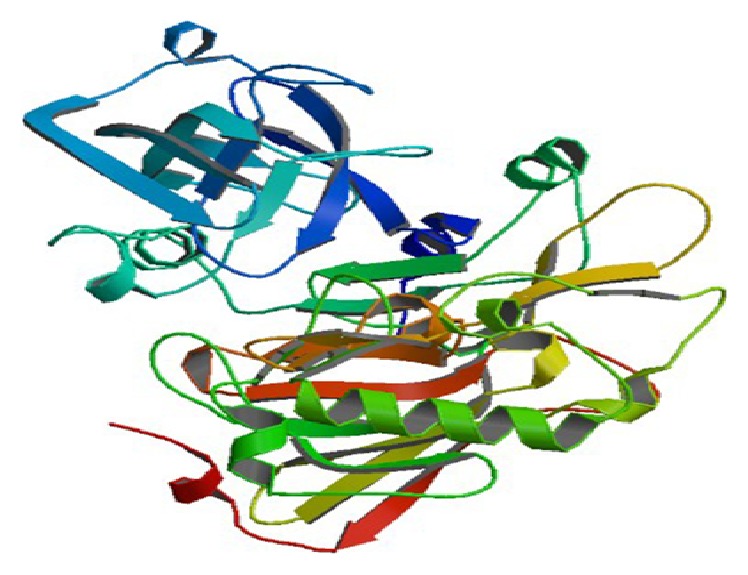
The target enzyme BACE 1 (memapsin 2,* PDB ID: 1SGZ*).

**Figure 4 fig4:**
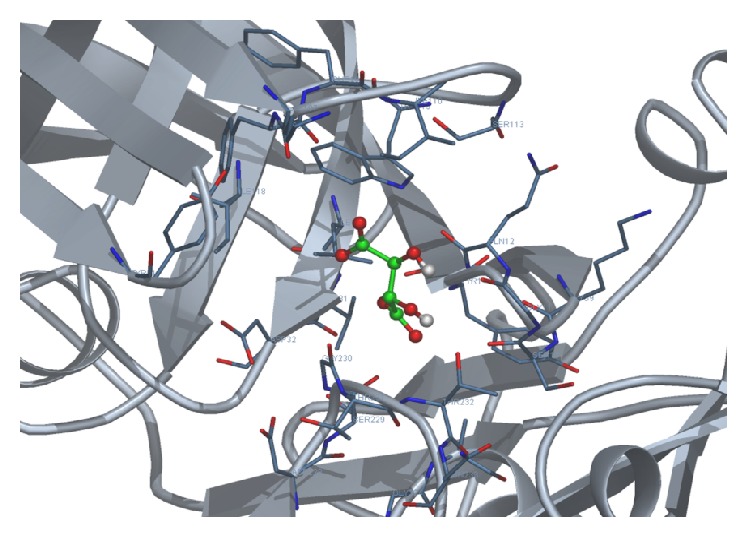
Docking of acylguanidine 7a with 1SGZ protein.

**Figure 5 fig5:**
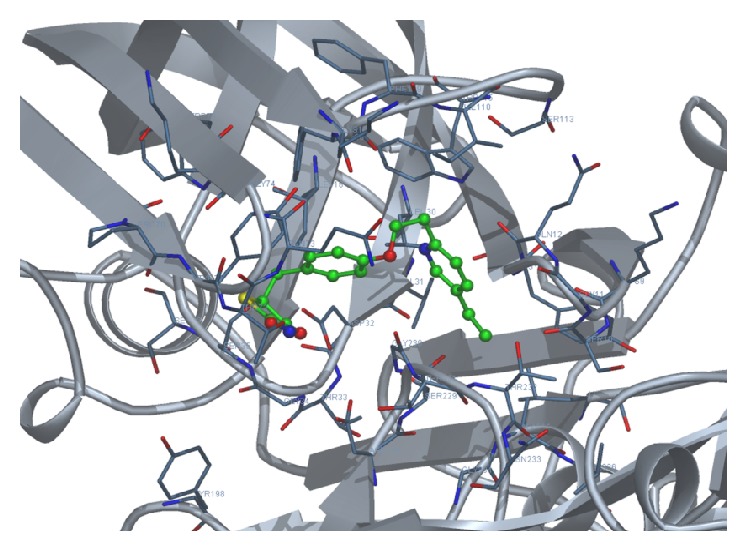
Docking of pioglitazone with 1SGZ protein.

**Figure 6 fig6:**
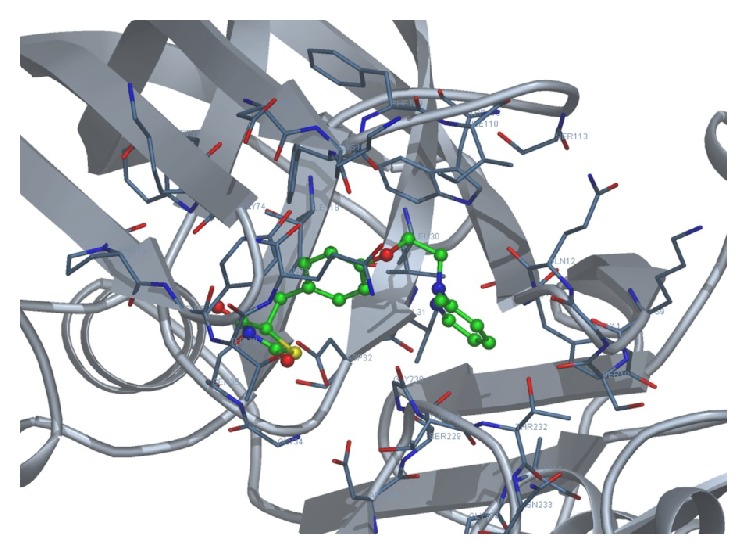
Docking of rosiglitazone with 1SGZ protein.

**Figure 7:**
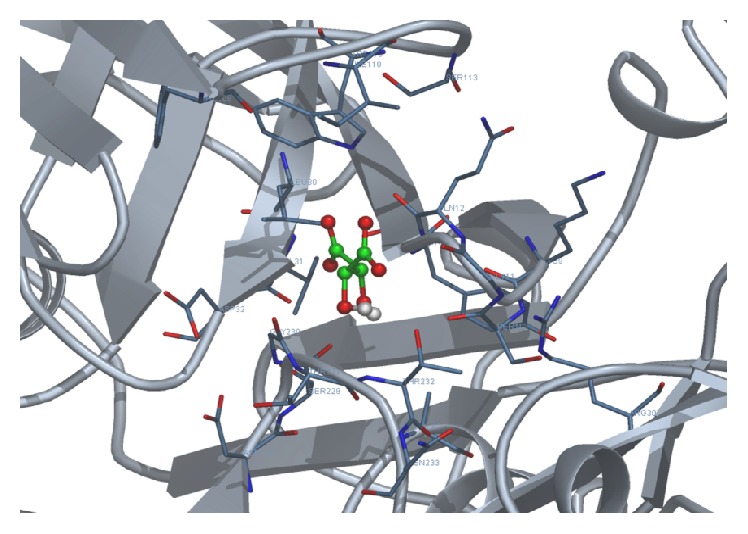
Docking of tartaric acid with 1SGZ protein.

We docked each of the four test ligands, namely, acylguanidine 7a, pioglitazone, rosiglitazone, and tartaric acid, with our target protein 1SGZ (*β*-secretase) separately by Docking Server. We found the following best results with each of the test ligands: Figures 3 [Fig fig4] [Fig fig5] [Fig fig6]–7.

### 3.1. Binding Energy

**Figure 8:**
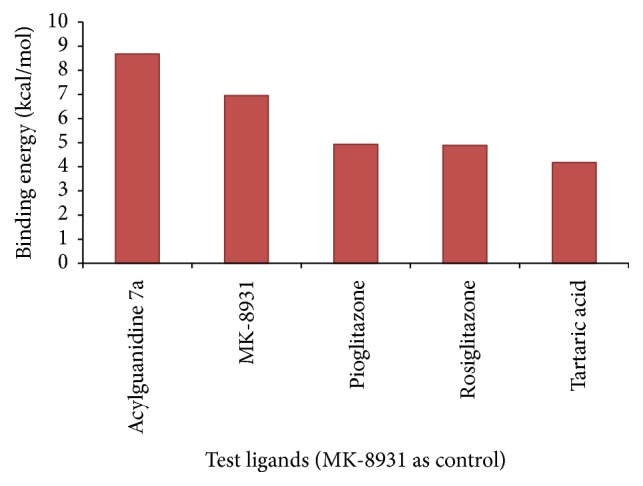
Binding energy values of the test ligands; MK-8931 is the study control.

Binding energy is the primary parameter which is generated as a result of molecular docking. It gives us the idea of strength and affinity of the interaction between the ligand and the receptor. The greater the binding energy is, the weaker the interaction is and vice versa. Thus during any docking study, we intend to look for the ligand which displays the least binding energy, thus the best affinity among the test molecules. Among the test candidates in this study, acylguanidine 7a displayed the lowest binding energy of −8.68 kcal/mol. The binding energy of the control MK-8931 was much higher than acylguanidine 7a, as found in our study; thus acylguanidine 7a displayed much better binding than the control molecule. The binding energies of the test ligands and the control have been depicted in Figure 8.

### 3.2. Drug-Likeness Prediction Studies

A good drug candidate is absorbed in required time and well distributed throughout the system for its effective metabolism and action. Toxicity is another very important factor which often overshadows the ADME behaviour. Failure of drugs at clinical trial stage due to adverse effects generated because of their toxicity proves very expensive and detrimental in the drug development process.* In silico* drug-likeness prediction along with further ADME/Tox tools presents an array of opportunities which help in accelerating the discovery of new targets and ultimately lead to compounds with predicted biological activity.  Table 1 depicts the drug-likeness properties of test compounds with least binding energies predicted using OSIRIS Property Explorer. The OSIRIS tool measures the *c*log⁡*P* value (logarithm of compound's partition coefficient between *n*-octanol and water) which is a well-established measure of the compound's hydrophilicity. Higher *c*log⁡*P* value indicates lower hydrophilicity and, thus, poor absorption and permeation. A log⁡*S* value indicates solubility; the lesser the log⁡*S* value, the higher the solubility which would enhance the absorption. A lower molecular weight would again enhance the absorption rate and thus most of the drugs are tried to be kept at the lowest possible molecular weight [15]. TPSA or* Topological Polar Surface Area* indicates the surface belonging to polar atoms in the compound. An increased TPSA is associated with diminished membrane permeability and compounds with higher TPSA were better substrates for p-glycoprotein (responsible for drug efflux from cell). Thus comparing the compounds, lower TPSA was favorable for drug-like property. It was also predicted that a molecule with better CNS penetration should have lower TPSA value [16, 17]. Of all the toxicological features predicted like mutagenicity, tumorigenicity, irritability, and reproductive toxicity, only tartaric acid was found to be quite toxic to reproductive system and all the test ligands were free of other advert properties [15]. One of the test ligands of this study, acylguanidine 7a, was shown to comply best with these properties used to predict drug-likeness (Table 1).

### 3.3. ADMET Prediction

**Figure 9:**
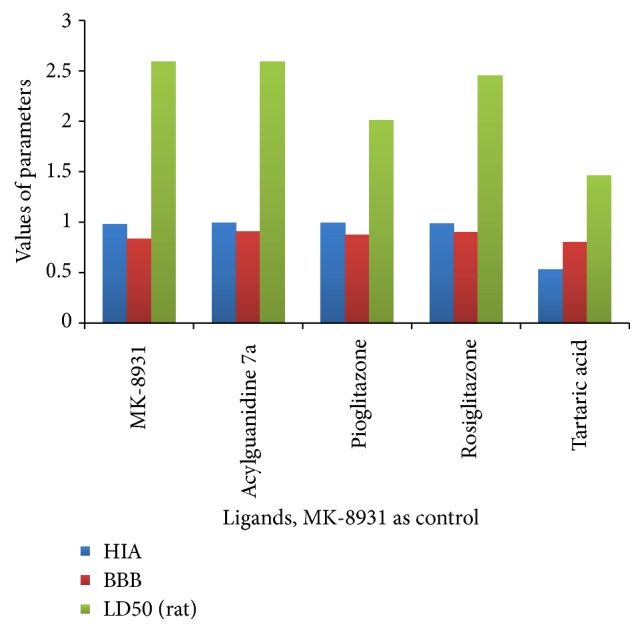
Comparative HIA, BBB, and LD50 of the test ligands and the control.

ADMET properties, as derived from admetSAR server, reveal that acylguanidine 7a and pioglitazone had better Human Intestinal Absorption (HIA) score than the control MK-8931. Greater HIA denotes that the compound could be better absorbed from the intestinal tract upon oral administration. The penetration through the Blood-Brain Barrier (BBB) came out to be best for acylguanidine and was significantly higher than the control molecule (0.9 versus 0.8, resp.). When it comes to predicting the efflux by P-glycoprotein (P-gp), acylguanidine comes out to be a substrate and noninhibitor of P-gp while pioglitazone came out as a nonsubstrate and noninhibitor of P-gp similar to our control molecule. Rosiglitazone was a substrate/noninhibitor while tartaric acid was a nonsubstrate/noninhibitor. In terms of metabolism, we found that acylguanidine was a nonsubstrate (but noninhibitor) of CYP450 microsomal enzyme while our control molecule was shown to be metabolized by CYP450 since it comes out to be a substrate and noninhibitor. A noninhibitor of CYP450 means that the molecule will not hamper the biotransformation of drugs metabolized by CYP450 enzyme. AMES toxicity test is employed to know whether a compound is mutagenic or not. Similar to the control MK-8931, all the test ligands displayed negative AMES toxicity test which means that the ligands are nonmutagenic. Carcinogenic profile also revealed that the ligands were noncarcinogenic similar to the control molecule. Acute oral toxicity was found to be highest for tartaric acid. All other test ligands and the control had low and almost similar oral toxicity. Important information obtained from admetSAR server was the computed LD50 dose in rat model. Comparing the LD50 doses, a compound with lower dose is more lethal than the compound having higher LD50. From our observation, we found that acylguanidine had almost the same LD50, compared to the control MK-8931 (2.58 versus 2.59, resp.). Tartaric acid had the lowest LD50 of 1.46 and was most toxic among the test ligands.  Table 2 illustrates the various ADMET parameters obtained from admetSAR tool.  Figure 9 depicts the comparative HIA, BBB, and LD50 values of the test ligands and the control.

## 4. Conclusions

Our admetSAR study revealed that, comparing and analyzing all the parameters, acylguanidine 7a could be projected as a potent BACE 1 inhibitor. Its ADMET properties displayed much similarity with our control MK-8931 which is already in the advanced clinical trial. Tartaric acid was found to be least suitable because of the lowest LD50 value apart from other parameters. In conclusion, the compound acylguanidine 7a, followed by pioglitazone, could prove to be remarkable base drug candidates as they are potent, selective, orally bioavailable, and nontoxic *β*-secretase inhibitors. In the present study, we identified that the best result of all the dockings in Docking Server was obtained between 1-SGZ (target protein) and acylguanidine 7a followed by MK-8931 (control ligand) and then pioglitazone (thiazolidinedione class) with respect to their free binding energy, whereas OSIRIS Property Explorer has shown tartaric acid, of all four test ligands, to have the highest toxicity in terms of reproductive effects and hence it is unfavorable as a drug candidate, as verified above too. The present study represents only the* in silico* docking of four different test ligand compounds against our target *β*-secretase enzyme and their computational analysis. These ligands could be used as base structure and different structure modifications could possibly bring more potent molecules. We could not proceed further with the* in vitro* and* in vivo* testing due to lack of the required facilities, and thus this study needs further* in vitro* and* in vivo* animal studies for development and authentication of these probable potent inhibitors of *β*-secretase for the treatment of Alzheimer's disease.

## Figures and Tables

**Table 1:** Drug-likeness prediction through OSIRIS Property Explorer.

S. number	Ligand	*c*log⁡*P*	Solubility log⁡*S*	Molecular weight	TPSA	Drug score
1	Acylguanidine 7a	2.16	−4.02	318	86.4	0.69
2	Pioglitazone	3.08	−3.84	356	93.59	0.76
3	Rosiglitazone	2.10	−3.67	357	96.83	0.8
4	Tartaric acid	−2.71	0.32	150	115	0.57

**Table 2:** ADMET profile of the test ligands and the control.

Compound	HIA	BBB	CYP inhibition/substrate	AMES toxicity	Carcinogenicity	LD50 in rat
MK-8931 (control)	0.9823	0.8354	Substrate/noninhibitor	Nontoxic	Noncarcinogenic	2.5901
Acylguanidine 7a	0.9916	0.9069	Nonsubstrate/noninhibitor	Nontoxic	Noncarcinogenic	2.5881
Pioglitazone	0.9952	0.8753	Substrate/inhibitor	Nontoxic	Noncarcinogenic	2.0115
Rosiglitazone	0.9861	0.8994	Substrate/inhibitor	Nontoxic	Noncarcinogenic	2.4515
Tartaric acid	0.5320	0.8035	Nonsubstrate/noninhibitor	Nontoxic	Noncarcinogenic	1.4627
